# Exploring behavioral outcomes of gonadectomy in pets: a need for personalized approaches

**DOI:** 10.1590/1984-3143-AR2025-0125

**Published:** 2026-07-06

**Authors:** Fabiano Borba Guimarães, Renata Oliveira Barreto, Maricy Apparício

**Affiliations:** 1 Departamento de Cirurgia Veterinária e Reprodução Animal, Faculdade de Medicina Veterinária e Zootecnia, Universidade Estadual Paulista “Júlio de Mesquita Filho” – UNESP, Botucatu, SP, Brasil

**Keywords:** castration, spay, canine, animal welfare, physiology, hypothalamic-pituitary-adrenal axis

## Abstract

Gonadectomy, the surgical removal of reproductive organs—is commonly practiced in canine management and is often associated with responsible pet ownership. Although advised for preventing unwanted litter and specific health hazards, its effects on canine behavior remain contentious and little studied. This article analyzes the physiological and behavioral effects of gonadectomy in dogs, emphasizing other sexually dimorphic behaviors including mounting, wandering, and urine marking, alongside aggression, fear, anxiety, separation-related issues, and canine cognitive dysfunction. Gonadectomy impacts hormonal equilibrium by modifying the hypothalamic-pituitary-adrenal and hypothalamic-pituitary-gonadal axes, hence affecting stress responses, cognition, and behavior. Certain research indicates that neutering diminishes urine marking and roaming; nevertheless, the results are inconsistent. Conflicting research suggests that gonadectomy may intensify aggression, fearfulness, and anxiety, thus undermining the human-animal bond. The effects of gonadectomy on cognitive impairment remain ambiguous. Alternative methods, including vasectomy, ovary-sparing spay, and chemical castration, provide reproductive regulation while maintaining hormonal function, potentially alleviating negative behavioral consequences. Due to the complex aspects of behavioral development, encompassing heredity, environment, and personal experiences, a general policy on gonadectomy is unsuitable. This review highlights the necessity of personalized assessments when advising gonadectomy for behavioral management in canines. Veterinarians must meticulously evaluate the advantages and disadvantages, considering the unique circumstances of each animal and seeking guidance from veterinary behaviorists, as necessary. Routine desexing should not be universally advocated only for the purpose of mitigating behavioral issues. Additional research is crucial to comprehend the intricate link between gonadectomy and canine behavior.

## Introduction

Gonadectomy, the surgical removal of gonads, is the most prevalent procedure conducted on dogs and is frequently linked to responsible pet management (Tadich et al., 2024). In addition to other reasons, this approach is based on the belief that reproductive behaviors contribute to issues such as aggression, abandonment, and animal transfers, with sexual behavior concerns being among the most reported by pet owners (Ben-Michael et al., 1997). Despite its widespread acceptance, the scientific exploration of neutering remains limited and under-investigated (Dehasse, 2024).

Neutering is often advised for multiple reasons such as preventing unwanted litters and reducing health risks, including prostate disorders, mammary tumors, and pyometra (Costa et al., 2022). It also triggers physiological, metabolic, endocrine, behavioral, and neoplastic changes in dogs (Kutzler, 2023). This review emphasizes behavior-related issues and their physiological bases, recognizing that undesirable behaviors can threaten the human-animal bond and sometimes lead to euthanasia, raising animal welfare concerns.

While neutering is widely applied, there is limited research on its behavioral effects, creating a knowledge gap in understanding its full implications. Studies suggest neutered dogs may have a 2.5-fold increased risk of mortality related to behavioral problems compared to intact dogs (Yu et al., 2021). To address canine welfare, it is important to investigate whether gonadectomy can mitigate behavioral issues. Throughout this review, terms like desexing, neutering, gonadectomy, and sterilization will be used interchangeably, with “castration” referring to males and “spaying” to females (McKenzie, 2010).

## Impacts of gonadectomy on reproductive physiology

**Figure 1:**
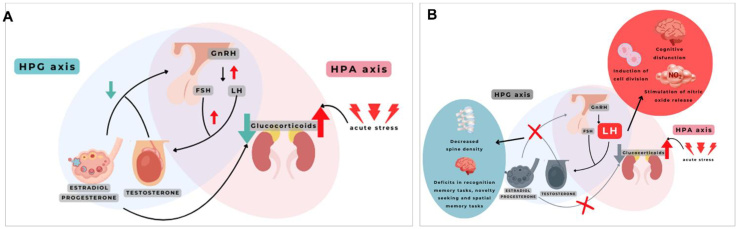
Representation of the connection between the HPG (hypothalamic-pituitary-gonadal) and HPA (hypothalamic-pituitary-adrenal) axis. (A) HPG and HPA axis in intact dogs. The interaction between GnRH (gonadotropin-releasing hormone), FSH (follicle-stimulating hormone), estradiol, testosterone, and glucocorticoids play a crucial role in hormone balance, brain function, and responses to acute stress; (B) Effects on gonadectomized dogs. Gonadectomy can cause disruption of hormonal pathways (red X’s), resulting in increased levels of LH and negatively affecting cognitive function and stress response mechanisms.

The influence of physiology on behavior primary involves two key axes: the hypothalamic-pituitary-adrenal (HPA) and hypothalamic-pituitary-gonadal (HPG) axes. The HPA axis, a conserved stress-response system in vertebrates, manages stress and maintains homeostasis in mammals (Sapolsky, 2021). The HPG axis, stimulated by the hypothalamus, produces gonadal hormones such as testosterone and estradiol that regulate HPA axis’s response to stress, with these hormones exerting activational and organizational effects on its function (Heck and Handa, 2019; Figure 1). Estradiol, mainly produced via aromatase, influence widespread brain areas, affecting mood, cognition, and memory -factors relevant to conditions like dementia (Ishikawa et al., 2006; McEwen, 2002).

In fully developed intact mammals, the hypothalamus triggers the anterior pituitary to secrete luteinizing hormone (LH) through gonadotropin-releasing hormone (GnRH) (Marques et al., 2022). In gonadectomized animals, the absence of steroid feedback causes LH levels to rise dramatically, with widespread effects in various tissues, potentially promoting cell division and nitric oxide release (Kutzler, 2023; Zwida and Kutzler, 2016). Elevated brain-derived LH has been linked to cognitive deficits, increased beta-amyloid plaques, and conditions resembling Alzheimer’s disease in older dogs, which show behavioral decline and brain pathology similar to humans (Blair et al., 2015; Mihevc and Majdic, 2019).

In females, hormonal fluctuations during estrus -marked by peaks and declines in estrogen and rises in progesterone – control of sexual receptivity behavior, with estrogen initiating and progesterone intensifying these changes (Apparício and Vicente, 2015). Progestagens, beyond their role in suppressing androgens, have a calming neurological effect and may reduce behavioral issues like aggression, although the detailed mechanisms are not fully understood (Knol and Egberink-Alink, 1989). Additionally, progesterone may help mitigate behavioral problems in intact females, further highlighting the complex hormonal regulation of canine behavior (Dinwoodie et al., 2019).

### Estrus and pseudocyesis

In female dogs, spaying is performed to suppress estrus and reproductive activities, including pseudocyesis (Root et al., 2018). Canine pseudocyesis, also known as pseudopregnancy, false pregnancy, or nervous lactation, is a common condition observed in non-pregnant females during late diestrus or early anestrus. It is characterized by varying degrees of mammary gland enlargement, maternal behavior, and lactation (Gobello, 2021). Pseudopregnancy may occasionally arise because of sterilization, especially in sensitive bitches that are spayed during diestrus (Root et al., 2018).

Furthermore, estrus influences certain behaviors in female dogs. The responsive posture exhibited by estrous bitches is a result of estrogen's effect on spinal neuronal tissue and cutaneous tactile sensory components (Hart, 1970).

## Impacts of gonadectomy on behavior

### Sexually dimorphic behavior

Sexually Dimorphic Behavior (SDB) refers to behaviors that are more pronounced in one sex, though not all sexual behaviors exhibit dimorphism (Kelley, 1988); for example, the play bow occurs similarly in both sexes and without sexual bias (Bekoff, 1974). Variations in cell quantity and gene expression, influenced by organizational and activational effects of sex hormones, contribute to neural differences underlying these behaviors. In females, sexual receptivity is regulated by progesterone receptors (PR), with neuronal ablation affecting both females and males differently, indicating that sex-specific neurons regulate behaviors in both genders (Yang et al., 2013). During estrus, behaviors like urine marking, roaming, and mounting increase (Roulaux et al., 2020), which enhances the female’s chances of reproductive success. These behaviors, like urine marking, are intended to communicate the female’s estrus status to males through pheromones, including methyl-p-hydroxybenzoate (Mertens, 2006).

The fetal nervous system has the potential to express sex-typical behaviors, and exposure to testosterone in utero can induce male-like behaviors in female dogs, such as urination postures and mounting; these behaviors can persist into adulthood without further hormonal influence (Beach, 1975). In males, testosterone regulates behaviors via the medial preoptic area and anterior hypothalamus (MPOA-AH), which are critical for sexual activity, with damage to these regions impairing mating behaviors and hormone implants restoring activity (Yahr, 1983). Testosterone also prepares the nervous system for future behaviors like marking, though it is not required to maintain them once developed (Hart, 1970; Ranson and Beach, 1985).

Gonadectomy is frequently recommended to reduce SDB such as urine marking, roaming, and mounting (Roulaux et al., 2023), with studies showing over a 50% reduction in these behaviors in more than 60% of dogs, and even greater improvements in some cases (Neilson et al., 1997; Kriese et al., 2022). However, conflicting findings exist; for instance, Zink et al. (2023) reported that gonadectomized males and females with ovary-sparing hysterectomy exhibited higher marking and mounting tendencies compared to intact or spayed females, with no clear behavioral difference between gonadectomized and intact males. Table 1 summarizes the possible outcomes of neutering on sexually dimorphic behavior (SDB) by category.

**Table 1:** Possible outcomes of neutering on sexual dimorphic behavior (SDB) by category.

**Behavior**	**Increased**	**Decreased**	**No Change**
**Mounting**	- Females undergoing OSH* and Intact males exhibited a higher propensity to mount compared to intact and spayed females (Zink et al., 2023)^5^	- Over 60% of dogs showed more than 50% improvement, with 25-40% demonstrating improvements exceeding 90% (Neilson et al., 1997)^5^- Mounting diminishes with greater PLGH** and aging (McGreevy et al., 2018;^5^Roulaux et al., 2020)^5^	- No significant correlation found (Garde et al., 2016)1
			- No substantial difference in mounting behaviors between gonadectomized and intact male dogs (Zink et al., 2023)^5^
**Roaming**	No data indicating an increase.	- Significant improvement of about 90% (Hart and Eckstein, 1997)3	
**Urine Marking**	- May increase during estrus (Roulaux et al., 2020)5	- Reduction in urination frequency and raised-leg posture ranging from <25% to 75% (McGuire, 2019)^4^	- No substantial difference in marking behaviors between gonadectomized and intact male dogs (Beach, 1974;2 Zink et al., 2023)^5^
			- Neutering does not significantly influence urine marking behavior in females (McGuire, 2019;4 Balogh et al., 2018)^5^

Notes: *OSH: ovary-sparing hysterectomy; **PLGH: Percentage of Lifetime Gonadal Hormones. Sample origin: ^1^free ranging; ^2^lab dogs; ^3^lab and owned (mixed data); ^4^shelters; ^5^owned dogs.

#### Mounting behavior

Mounting, which is defined as the act of holding another dog between the front legs—typically from behind but occasionally from the front (Kaufmann et al., 2017)—is commonly observed in male dogs and can often be reduced through desexing (Palestrini et al., 2021; Hart and Eckstein, 1997). However, mounting behavior can arise from various factors beyond sexual behavior, including play and exploratory actions. In some instances, mounting can also lead to sexual excitement and frictional movements (Mertens, 2006). Understanding these underlying factors is crucial, as mounting may be associated with stress or anxiety—exemplified by human-directed mounting — or it can manifest as playful activity reflecting normal social behavior and elevated arousal levels in dogs (Roulaux et al., 2020).

When considering gonadectomy as a solution for addressing mounting behavior, it is crucial to factor in previous sexual experience. Urfer and Kaeberlein, (2019) reference Beach (1970) to emphasize that increased copulatory experience in male dogs may diminish the effectiveness of neutering in addressing this behavior. Beach's research on the long-term effects of castration indicates that prior sexual experience may mitigate the impact of neutering on reducing mounting behavior.

Dogs with a greater percentage of lifetime exposure to gonadal hormones (PLGH) generally exhibit less mounting behavior (McGreevy et al., 2018). McGreevy’s observations primarily focused on mounting objects, such as furniture, and people, rather than other dogs. However, intact dogs may also exhibit mounting behavior directed at other dogs, which could introduce a bias by potentially underestimating or misrepresenting the total frequency of mounting behavior. This discrepancy highlights the need for more nuanced data collection that accounts for different targets of mounting behavior. Additionally, mounting behavior typically decreases with age, suggesting that neuter status may not be the primary factor contributing to the decline in this activity (Roulaux et al., 2020).

Interestingly, surgically castrated free-roaming dogs have shown no decrease in sexual behavior. Garde et al., (2016) found no significant correlation between serum testosterone levels and the display of courting behaviors, including mounting, pursuing another dog, and reacting to threats from other dogs. Similarly, Hopkins et al. (1976, as cited in Palestrini et al., 2021) did not observe behavioral changes in the neutered group. Since this group was monitored for approximately 4-5 months, they suggested that a minimum of six months is necessary for behavioral changes to manifest following castration. Moreover, mounting may serve as a displacement behavior—defined as a typical action performed in an inappropriate context (Falk, 1977)—and may not necessarily be linked to sexual motivation.

#### Roaming behavior

Roaming is not exclusively sexual behavior; it is also motivated by the need for exercise and exploration (Kobelt et al., 2003). The underlying reasons for wandering can influence the effects of desexing. Research involving owned dogs suggests a positive relationship between desexing and a reduction in roaming behavior (Roulaux et al., 2020). For instance, Hopkins et al. (1976 as cited in Hart and Eckstein, 1997) reported that neutering owned dogs resulted in a 90% decrease in roaming.

#### Urine marking behavior

Androgens regulate urine marking by stimulating the MPOA-AH and its projections to the medial forebrain bundle. These regions are crucial for urine marking to occur and facilitate the androgenic influence on this behavior (Yahr, 1983). Sex hormones significantly influence early-life organizational effects, which can complicate expectations regarding the consequences of castration in adult animals (Roulaux et al., 2020).

Beach (1974) found that exposure to testosterone from birth is essential for developing the adult male pattern of frequent urine marking on vertical surfaces, utilizing the raised-leg stance. He observed no significant differences in the frequency of urination or the use of the raised-leg posture between intact adult males and those castrated in adulthood. This suggests that while testosterone may be essential for initiating adult male urine marking behavior, it may be less vital for its subsequent maintenance (Roulaux et al., 2020). Nevertheless, other studies indicate that certain shelter dogs neutered after reaching adulthood exhibit reductions in urination frequency and the adoption of the raised-leg stance, despite the continued presence of sexual dimorphism in this behavior (McGuire, 2019).

Urine marking is a sexually dimorphic behavior predominantly observed in intact adult male dogs, exhibiting notable differences when compared to intact females and castrated males. Male urine marking is characterized by two primary traits: the adoption of a raised-leg stance and the habitual marking of vertical or conspicuous surfaces. In contrast, females generally adopt a squatting position and urinate infrequently—primarily for bladder emptying—except during estrus (Hart, 1974). Neutering usually reduces urine marking in male animals; castrated males exhibit lower urination rates compared to intact males, with the extent of this reduction ranging from less than 25% to 75% (McGuire, 2019). However, castrated males still mark more frequently than both spayed and intact females.

Spaying, on the other hand, does not significantly affect urine marking behavior in females, as spayed and intact females demonstrate comparable urination rates. During 20-minute observations, intact females urinated 0.14 ± 0.16 times, compared to 0.16 ± 0.12 times for spayed females (McGuire, 2019). The urination behavior of intact female Labrador Retrievers, whether spayed before or after puberty, remained consistent and was unaffected by age, duration of ovarian exposure, or time elapsed since spaying, indicating no signs of masculinization in the absence of gonads. Due to the variability in behavioral outcomes associated with spaying across different dog breeds, caution should be exercised when generalizing these results from female Labrador Retrievers to other breeds (Balogh et al., 2018).

### Aggression

Aggression in animals is a social behavior crucial for resource acquisition, self-defense and the protection of one's group. It is facilitated by innate neural pathways and shaped by an animal's internal condition and prior experiences (Lischinsky and Lin, 2020). In dogs, aggression and disobedience pose significant risks for abandonment and euthanasia (Yu et al., 2021). Aggressive behavior can be categorized into three forms: (i) aggression directed towards familiar individuals, (ii) aggression towards strangers, and (iii) aggression towards other canines (Farhoody et al., 2018).

The impact of neutering on canine aggression is a highly debated topic in scientific literature. Canine aggression encompasses both hormonal and behavioral dimensions and cannot be exclusively mitigated through sterilization (Romagnoli et al., 2023). Research suggests that castration may reduce inter-male aggression, with findings indicating that aggression towards other animals often decreases after neutering (Kriese et al., 2022; Romagnoli et al., 2024). However, castration may not affect territorial or fear-induced aggression (Podberscek and Serpell, 1996). Conflicting findings have been recorded in some studies (Kaufman and Lapauw, 2020).

Conversely, recent evidence suggests that sterilization may intensify aggressive behavior towards humans or other family dogs (Li et al., 2025). For example, Hart et al. (2023) noted increased hostility in neutered males. Moreover, premature castration has been associated with a higher likelihood of aggressive behavior toward family members (Yu et al., 2021). English Cocker and Springer Spaniels females are more likely to be aggressive to their owners. Female aggressiveness also tends to increase when spaying is performed in bitches that were already aggressive (Romagnoli et al., 2024). Farhoody et al. (2018) documented a 26% increase in aggression towards strangers in dogs that were neutered between 7 and 12 months of age. Castrated males also tend to exhibit less emotional stability in stressful situations, which can lead to an increased propensity for aggression towards both familiar and unfamiliar individuals (Kaufmann et al., 2017).

In a study conducted at the Korean Air Force Dog Training Center, German Shepherd bitches aged 5 to 10 months that underwent ovariohysterectomy exhibited increased barking due to territorial aggression towards unfamiliar individuals. The spayed group barked an average of 45 times, while the control group averaged 26 barks, with the vocal pitch being significantly lower in the spayed group (Kim et al., 2005).

Some studies indicate that intact dogs tend to exhibit higher aggression ratings than spayed or neutered dogs, although the effect size is small (McGuire and Song, 2023). Moreover, research by Blackwell et al., (2008) and Hsu and Sun, (2010) found no significant impact of neuter status on the overall incidence of undesired behaviors, including aggression, but they did not specify the types of aggression affected.

The age at which a dog undergoes gonadectomy significantly influences behavioral development. Dogs neutered at a young age may display increased aggression, particularly towards unfamiliar individuals and other dogs, especially those with a low Percentage of Lifetime Exposure to Gonadal Hormones (PLGH) score (McGreevy et al., 2018; Riemer et al., 2021) One hypothesis suggests that testosterone may play a protective role against fearfulness in aging canines; thus, neutering could negatively impact a dogs' coping mechanisms—behaviors used to manage internal and external stressors (Algorani and Gupta, 2023)— resulting in heightened aggression (McGreevy et al., 2018; Riemer et al., 2021).

Gonadectomized male dogs often display increased aggression towards trainers, while intact-trained male and female dogs exhibit reduced biting and aggression (Fattah and El Abdel-Hamid, 2020). Castrated male dogs may also receive heightened attention from intact dogs in the anal region (Kaufmann et al., 2017). Castration may disrupt a dog’s communication, exacerbating nervousness and insecurity. This can signal a heightened emotional state to other dogs, potentially leading to increased aggressive behavior. A digital survey indicated that 92% of dog bites originated from neutered animals, which aligns with the fact that most of the sampled population was neutered (84% of males were castrated, and 85% of females were spayed) (Dinwoodie et al., 2019). Associations were identified between male sex and biting (OR = 1.37; 95% CI: 1.11–1.70; n = 4100; p = 0.003) as well as between owner-directed aggression and male sex (OR = 1.85; 95% CI: 1.45–2.36; n = 4100; p < 10^−6^). Nevertheless, neutering was also correlated with aggression (OR = 2.64; 95% CI: 1.83–4.26; n = 4096; p = 10^−6^), as 36% of castrated males displayed aggressive behavior compared to 20% of intact males, and aggression rates increased from 16% in intact females to 29% in spayed females.

The incidence of dog bites can vary based on factors beyond neuter status. For instance, in a comparison of two regions—San Francisco and Kingston—56% of aggressive dogs in San Francisco were neutered, whereas only 7% were neutered in Kingston, indicating that geographical factors may influence the relationship between neutering and aggression (Messam et al., 2012). Table 2 summarizes the possible outcomes of neutering on sexually dymorphic behavior (SDB) by category.

**Table 2:** Possible outcomes of neutering on aggression.

**Increased**	**Decreased**	**No Change**
**Towards familiar individuals**		
Increased likelihood of aggression against family members (Yu et al., 2021)^3^. Tendency for aggressive behavior towards family (Kaufmann et al., 2017)^3^. Heightened aggression towards trainers (Fattah and El Abdel-Hamid, 2020)^3^		
Towards strangers		
26% increase in aggression towards strangers (Farhoody et al., 2018)^1^	Spayed or neutered dogs exhibited lower ratings of aggression compared to intact dogs (McGuire and Song, 2023)2	
Tendency for aggressive behavior towards new people (Kaufmann et al., 2017)^3^		
Increase in territorial aggression towards unfamiliar individuals (Kim et al., 2005)1		Gonadectomy may not influence territorial or fear-induced aggression (Podberscek and Serpell, 1996)^3^
Decreased PLGH* increases aggression towards unfamiliar individuals (McGreevy et al., 2018)^3^		
Towards other animals		
Tendency for aggressive behavior towards conspecifics (Kaufmann et al., 2017)^3^	Diminish inter-male aggressiveness (Kriese et al., 2022)^3^	
Decreased PLGH increases aggression towards other canines (McGreevy et al., 2018)^3^	Intact canines demonstrate increased aggressiveness relative to their desexed counterparts (McGreevy et al., 2018)3	

Notes: *PLGH: Percentage of Lifetime Gonadal Hormones. Sample origin: ^1^working dogs; ^2^shelters; ^3^owned dogs.

### Fear and anxiety

Fear and anxiety are significant emotional motivators, arising from the perception of real danger (fear) or potential threats (anxiety) that can jeopardize an individual's well-being (Boissy, 1995). Various factors, including early-life experiences, socialization, physical activity, dietary habits, genetic predispositions, environmental influences, and management strategies, can affect anxiety levels in dogs (Tiira and Lohi, 2015). Table 3 summarizes the possible outcomes of neutering on fear and anxiety.

**Table 3:** Possible outcomes of neutering dogs on fear and anxiety.

**Behavior**	**Increased**	**Decreased**
**Impulsive Response**	Previous fearfulness or anxiety can lead to more overt and impulsive responses to fear, including aggression (Storengen et al., 2014;^1^McGreevy et al., 2018)^1^	
**Boldness**		Decreased boldness (McGreevy et al., 2018)^1^
**Arousal and Nervousness**	Increased in male dogs (Fattah and El Abdel-Hamid, 2020)^1^	
**Confidence**		Reduced in male dogs (Fattah and El Abdel-Hamid, 2020)^1^
**Noise Phobia**	Increased; higher PLGH* diminishes reactions. Enhanced fear of storms, gunshots, loud noises (McGreevy et al., 2018;^1^Kutzler, 2020b)^2^	
**Biting**	Increased fear biting (Kutzler, 2020b)^2^	
**Shyness**	Increased (Kutzler, 2020b)2	
**Separation Anxiety**	Increased (Kutzler, 2020b)^2^	
**Submissive Urination**	Increased (Kutzler, 2020b)^2^	
**Reaction to Unexpected Objects**	Increased; higher PLGH diminishes reactions (McGreevy et al., 2018)1	
**Nail Trimming**	Increased; higher PLGH diminishes reactions (McGreevy et al., 2018)^1^	

Notes: *PLGH: Percentage of Lifetime Gonadal Hormones. Sample origin: ^1^owned dogs; ^2^mixed data.

Since boldness is correlated with reduced fearfulness and greater friendliness towards both humans and other dogs (McGreevy et al., 2018), gonadectomy may exacerbate behavioral issues associated with decreased boldness (Li et al., 2025). Spaying or castrating dogs with already exhibit fearfulness or anxiety is unlikely to provide favorable results due to the close relationship between fear and aggression (Storengen et al., 2014; McGreevy et al., 2018). Furthermore, because progesterone promotes calmness in female dogs, ovariectomy may increase their vulnerability to anxiety disorders (Meneses et al., 2021).

Increasing the Percentage of Lifetime Exposure to Gonadal Hormones (PLGH) may help protect male dogs from fear-related behaviors. Higher PLGH levels have been associated with reduced reactions to sudden or loud noises, unexpected objects, nail trimming, and various other anxiety-inducing situations (McGreevy et al., 2018). Additionally, owners of spayed dogs have reported a greater frequency or intensity of panic responses to loud sounds, unfamiliar items encroaching on sidewalks, and interactions with unfamiliar dogs exhibiting barking, growling, or jumping behaviors (Balogh et al., 2018). This suggests that, contrary to popular belief, gonadectomy does not necessarily result in a more behaviorally stable dog.

Neutering has been linked to increased arousal, nervousness, and decreased confidence in male dogs, making intact working dogs appear more composed and amenable (Fattah and El Abdel-Hamid, 2020). This finding emerged from a study of working dogs, who experience markedly different environments compared to companion dogs. Moreover, elevated testosterone levels are associated with reduced fear behaviors in animals (Tong et al., 2019), and increased fear resulting from gonadectomy in dogs may lead to increased aggression (Roulaux et al., 2020, Romagnoli et al., 2024). Additionally, testosterone therapy in a castrated male dog has shown a reduction in fear and avoidance of unfamiliar individuals (Brent et al., 2021), although further research with larger samples is needed.

Overall, gonadectomy may negatively impact various fear-related issues, such as fear of storms, gunshots, loud sounds, fear biting, shyness, separation anxiety, and submissive urination (Kutzler, 2020b).

### Separation related problems (SRPs)

Separation-related problems (SRPs) refer to behaviors that dogs exhibit when separated from their owners. This term encompasses various interchangeable terms in the literature, including separation anxiety, separation reactions, separation-related distress, separation anxiety syndrome, and separation anxiety disorders (Assis et al., 2020) . SRPs are complex conditions influenced by environmental, genetic, and learning factors, characterized by similar signs yet lacking a well-defined underlying cause (Assis et al., 2020).

Anxiety is not the sole underlying cause of SRPs; dogs may respond to their owner's absence due to a range of internal states, including fear and panic (Fattah and El Abdel-Hamid, 2020; Lenkei et al., 2021). Furthermore, discussions have explored the potential impact of excessive attachment or varying forms of connection between dogs and their owners in the development of SRP (Storengen et al., 2014). Consequently, the term “hyperattachment” to humans may not accurately describe the emotions associated with SRPs; rather, the type of attachment can differ between dogs with and without separation issues (Parthasarathy and Crowell-Davis, 2006).

The impact of gonadectomy on SRPs remains contentious - desexing may either worsen or alleviate these problems (Storengen et al., 2014). McGreevy et al. (2018) found that vocalizations, such as wailing while isolated, correlated with a reduced percentage of lifetime exposure to gonadal hormones (PLGH), suggesting that gonadectomy may exacerbate SRPs. In contrast, Spain et al. (2004) reported that separation anxiety diminished in dogs neutered before one year of age, indicating that early neutering might mitigate these issues. Also, a recent study showed that intact and younger dogs with young owners may exhibit more severe SRB (Sungkatavat et al., 2025).

Despite these contradictory findings, a correlation appears to exist between SRPs and sexual hormones, potentially affecting the regulation of fear. Early sterilization has been positively associated with fear and anxiety-related issues (Amat et al., 2020). Additionally, gonadectomized animals show a 20% likelihood of exhibiting SRPs, compared to 10% in the general population and 8.4% in a convenience sample analyzed at a behavioral clinic (Storengen et al., 2014).

### Canine cognitive dysfunction

Canine cognitive dysfunction (CCD) is a syndrome that affects senior dogs and is characterized by behavioral changes such as confusion, altered social interactions, sleep disturbances, inappropriate elimination, and fluctuations in activity levels (Landsberg et al., 2012). The relationship between gonadectomy and CCD is complex and not fully understood. Research suggests that gonadectomized dogs have a higher susceptibility to CCD; specifically, neutered dogs are 2.3 times more likely to develop CCD compared to intact dogs (Benjanirut et al., 2018). Neutered males may face an increased risk of progressing from mild to severe cognitive impairment, potentially due to testosterone’s role in mitigating cognitive decline (Hart, 2001). Aging is a known risk factor for this disorder and may function as a confounding variable, given that gonadectomized dogs tend to have longer lifespans (Hoffman et al., 2013).

The hypothesized protective role of estrogen in intact females remains uncertain (Hart, 2001). Scandurra et al. (2019) found that intact female dogs performed better in following human pointing gestures than spayed females. Intact females were more likely to adhere to pointing cues quickly and accurately, while gonadectomized females exhibited a significantly higher incidence of no-choice responses. These findings suggest that ovariectomy may negatively impact dogs' socio-cognitive abilities, particularly their responsiveness to human gestures. Moreover, age-related declines in estrogen levels can impair cognition in humans, primarily due to reduced dendritic spine density and synaptic changes in the prefrontal cortex and hippocampus, leading to deficits in recognition memory, novelty-seeking, and spatial memory (Blair et al., 2015). In the human male, the relationship between reduced testosterone and cognition is still debated, highlighting the need for further investigation (Kaufman and Lapauw, 2020).

However, alternative studies present conflicting evidence. MacQuiddy et al. (2022) reported the highest prevalence of CCD in intact females, indicating that females, in general, had a greater prevalence than males. Additionally, Katina et al. (2016) found no significant correlation between neuter status and the onset of CCD. Both MacQuiddy et al. (2022) and Katina et al. (2016) recognized that the uneven distribution of surveyed dogs might have influenced their results. Consequently, the relationship between neuter status and CCD remains ambiguous, highlighting the need for further research with more balanced study groups.

### Alternatives to canine neutering

Ovary-sparing hysterectomy (OSH) and vasectomy are procedures that preserve endocrine function while suppressing reproduction, often with fewer side effects than traditional gonadectomy. OSH removes the uterus and cervix while keeping functional ovaries, whereas vasectomy excises or obstructs the vas deferens to prevent sperm release, rendering the dog sterile (Kutzler, 2020a).

Research conducted by Zink et al. (2023) found that 53% of castrated dogs exhibited problematic behavior (aggression, anxiety-based behaviors, extreme fears, and nuisance behaviors-urine marking and mounting behavior), compared to 35% of intact males, 28% of intact females, 41% of spayed females, and 43% of dogs that underwent OSH. Although OSH and spayed females demonstrated similar rates of problematic behavior, the authors concluded that “[…] the prevalence of both problematic and nuisance behaviors diminished with increased exposure to gonadal hormones”.

Another alternative is chemical castration. Some studies suggest that chemical castration may not have the same behavioral effects as surgical castration; however, these investigations focused primarily on working dogs (Gfrerer et al., 2019). Following chemical castration, male dogs may exhibit increased reproductive behavior for 1 to 3 weeks post-treatment (Romagnoli et al., 2023). In one study, dogs treated with chemical castration exhibited behavioral profiles—aggression, fear/insecurity, and play—that were comparable to those reported for surgically neutered dogs (Steur, 2010). Although chemical castration is not widely available and is primarily used for male fertility control, its similar behavioral outcomes provide a valuable point of comparison when discussing neutering options (Romagnoli et al., 2024; Roulaux and van Herwijnen, 2026).

## Discussion

While the concept of "responsible ownership" is often promoted—with occasional recommendations for mandatory neutering in most cases (Yu et al., 2021)—it is essential to customize intervention strategies to the complex dynamics of different dog populations, such as unowned dogs, roaming dogs, community-owned dogs, and shelter populations. This approach departs from one-size fits all suggestions, indicating that a uniform policy may not be the most effective solution.

The precautionary principle emphasizes the need for prudent actions to prevent significant and credible risks. The rationale behind a response depends on factors such as the balance between benefits and harm, realism, proportionality, and consistency (Resnik, 2004). Proportionality entails ensuring that measures taken to avert or mitigate a potential threat are commensurate with the severity and likelihood of that threat. Resnik argues that consistency enhances credibility by ensuring that new theories do not conflict with established knowledge unless supported by convincing evidence.

When applying the precautionary principle to dog neutering, consistency requires evaluating whether the potential hazards or threats associated with neutering align with established scientific facts and hypotheses regarding canine health and behavior. The precautions taken must be proportionate to the level of risk involved and should not be excessive. Veterinarians should weigh the reduction in certain diseases associated with dog neutering against the increased threats of behavioral disorders and other health complications, considering the unique circumstances of each individual case.

This evaluation may include other alternatives, including vasectomy or ovary-sparing hysterectomy. Specialists might opt for chemical castration to evaluate the behavioral effects of neutering prior to the surgical procedure. However, it is important to note that chemical castration is not widely available and primarily addresses male fertility.

Routine desexing may not consistently produce the desired behavioral changes, raising ethical concerns (Roulaux et al., 2020). Given the limited research in this area and the potential for unforeseen, often negative outcomes following gonadectomy, the veterinary community should avoid advocating for desexing solely to address behavioral issues. That said, if alternative interventions fail to resolve significant problems threatening the human-animal bond, gonadectomy may be considered as a supplementary treatment option.

While gonadectomy is frequently recommended to mitigate sexually dimorphic behaviors such as mounting, roaming, and urine marking, the effectiveness of this procedure can vary widely. Factors such as prior sexual experience, age at the time of castration, and individual behavioral incentives significantly influence outcomes. Moreover, conflicting evidence within the literature emphasizes the complexity of hormonal impacts on behavior, underscoring the need for personalized assessments when considering gonadectomy for behavioral control in dogs.

## Conclusion

Surgical sterilization should not be regarded as essential for responsible pet ownership, given the limited and conflicting evidence on its behavioral effects. Decisions should be tailored to each family’s specific context, prioritizing the animals’ well-being and considering alternative approaches. Consulting a veterinary and exploring all options, including less invasive procedures, is highly recommended before opting for sterilization as a behavioral intervention.

## Data Availability

No research data was used.
